# A Modified Triaxial Electrospinning for a High Drug Encapsulation Efficiency of Curcumin in Ethylcellulose

**DOI:** 10.3390/pharmaceutics17091152

**Published:** 2025-09-02

**Authors:** Xingjian Yang, Qiling Wang, Zhirun Zhu, Yi Lu, Hui Liu, Deng-Guang Yu, Sim-Wan Annie Bligh

**Affiliations:** 1 School of Materials and Chemistry, University of Shanghai for Science and Technology, 516 Jungong Road, Shanghai 200093, China; 2235051328@st.usst.edu.cn (X.Y.); huiliu@usst.edu.cn (H.L.); 2 Shanghai Experimental School, 300 Dongming Road, Shanghai 200125, China; 3 School of Health Sciences, Saint Francis University, Hong Kong 999077, China

**Keywords:** drug encapsulation efficiency, modified triaxial electrospinning, sustained release, core–sheath nanostructures, spinnable fluid

## Abstract

**Background:** Although electrohydrodynamic atomization (EHDA) consistently provides drug-encapsulation efficiencies (DEE) far above those of conventional bottom-up nanotechnologies, the question of how to systematically push that efficiency even higher remains largely unexplored. **Methods:** This study introduces a modified triaxial electrospinning protocol tailored to the application and benchmarks it against two conventional techniques: single-fluid blending and coaxial electrospinning. Ethylcellulose (EC) served as the polymeric matrix, while curcumin (Cur) was chosen as the model drug. In the triaxial setup, an electrospinnable, drug-free EC solution was introduced as an intermediate sheath to act as a molecular barrier, preventing Cur diffusion from the core fluid. Ethanol alone was used as the outermost fluid to guarantee a stable and continuous jet. **Results**: This strategy provided a DEE value of 98.74 ± 6.45%, significantly higher than the 93.74 ± 5.39% achieved by coaxial electrospinning and the 88.63 ± 7.36% obtained with simple blending. Sustained-release testing revealed the same rank order: triaxial fibers released Cur the most slowly and exhibited the smallest initial burst release effect, followed by coaxial and then blended fibers. Mechanistic models for both fiber production and drug release are proposed to clarify how the tri-layer core–shell structure translates into superior performance. **Conclusions**: The modified triaxial electrospinning was able to open a new practical route to produce core-sheath nanofibers. These nanofibers could provide a higher DEE and a better sustained drug release profile than those from the coaxial and blending processes.

## 1. Introduction

To a certain extent, the history of drug delivery is a joint history of pharmaceutical excipients and material conversion techniques [1,2,3,4,5]. New techniques are continuously introduced into this interdisciplinary field to unlock their potential for creating novel drug delivery systems [6,7,8,9]. In this nano era, almost all kinds of nanofabrication techniques are finding their way into nanopharmaceutical methods [10,11]. One of the most prominent examples is electrospinning [12,13,14,15].

Electrospinning—initially a textile technique alongside wet spinning, dry spinning, and other spinning processes that rely on mechanical forces—is becoming increasingly popular for producing a wide variety of medicated nanofibers [16,17,18,19,20,21]. It is generally regarded as an electrohydrodynamic atomization (EHDA) method, together with electrospraying and e-jet printing [22,23,24]. EHDA techniques, taking advantage of the facile interactions between electrostatic energy and working fluids, are advancing rapidly. One reason is that they readily accommodate advances in nanoscience and nanotechnology for creating complex nanostructures [25,26]. The other is the expanding capability of EHDA methods to process a wider range of materials more easily. In line with these two aspects, triaxial electrospinning (short for tri-layer coaxial electrospinning) is receiving increasing attention for creating tri-layer core–sheath nanofibers [27,28]. On the other hand, an increasing number of initially unspinnable fluids are being combined with electrospinnable solutions to enable new electrospinning processes and produce novel nanofibrous types [29,30,31].

Drug encapsulation efficiency (DEE) is always a major concern in developing novel nano-drug delivery systems [32,33]. Low DEE inevitably entails tremendous waste of costly active ingredients. EHDA methods offer advantages over traditional bottom-up synthesis techniques because they rely primarily on a physical drying process that proceeds rapidly under ambient conditions [34]. Consequently, EHDA products often exhibit high DEE values. In contrast, bottom-up nanofabrication methods often yield low DEE values; for example, molecular self-assembly can achieve only 6.5% [35]. This implies that more than 90% of the valuable active pharmaceutical ingredient is lost during material conversion. Because EHDA methods typically achieve higher DEE values than other nanofabrication processes, the question of how to further elevate DEE in EHDA has been largely overlooked, and few systematic investigations have been conducted at the intersection of electrospinning and drug delivery. Moreover, significant controversy exists in the literature regarding reported DEE values. Some publications have reported DEE values of 100%, attributing the result to the simple physical drying process inherent to electrospinning. Conversely, others have reported markedly lower measured DEE values, for example, only 22% [36]. Most recently, Deng et al. demonstrated that both 100% and lower DEE values are possible [37]. They prepared core–sheath nanofibers with TiO_2_ nanoparticles in the sheath and curcumin in the core to elucidate the origins of DEE variations in electrospun nanofibers, confirming that molecular diffusion between working fluids occurs during electrospinning [37]. However, the study did not systematically compare different electrospinning processes or provide clear guidance on how to intentionally achieve high DEE values through parameter manipulation.

Based on the foregoing discussion, we hypothesized that a modified triaxial electrospinning strategy could be exploited to ensure high DEE values. The strategy involves using an electrospinnable, drug-free polymer solution as the middle fluid to prevent diffusion of the core fluid into the environment and employing pure ethanol as the outer fluid to ensure continuous and robust nanofabrication. Curcumin (Cur), an active biomolecule, was selected as a model drug both for its widespread use in functional nanomaterials for food, drug delivery, and biomedical engineering applications [38], and for its strong yellow fluorescence, which allows easy visualization with the naked eye or under a camera’s magnesium lamp. As a natural active ingredient extracted from the root and stem of turmeric, Cur belongs to the polyphenolic compounds, having functions of anti-inflammatory, antioxidant, supporting liver metabolism, and promoting digestion. It may also have certain auxiliary effects on cardiovascular and cerebrovascular health, cognitive function, and cancer prevention. High DEE and sustained release are beneficial for its functional performances [39,40]. Ethylcellulose (EC), a cellulose derivative, is an inert and widely used polymeric excipient for sustained drug release [41,42,43]. Here, it was employed as a model pharmaceutical polymeric excipient. 

## 2. Materials and Methods

### 2.1. Materials

Ethylcellulose (EC, viscosity 6–9 mPa·s, CAS No. 9004-57-3) was sourced from Aladdin Chemistry Co., Ltd. (Shanghai, China). Curcumin (Cur, CAS No. 458-37-7), dichloromethane (DCM, CAS No. 75-09-2), and anhydrous ethanol (CAS No. 64-17-5) were purchased from Sigma-Aldrich (Shanghai, China). Phosphate Buffer Solution (PBS, 0.1 M, pH 7.0) was purchased from Tianjin Zhiyuan Chemical Reagent Co., Ltd. (Tianjin, China). Water was double-distilled immediately before use. All chemicals were used as received without further purification.

### 2.2. Electrospinning

A laboratory-scale electrospinning system was constructed to prepare nanofibers, the key feature of which was a custom-designed, detachable triaxial eccentric spinneret. The setup comprised three syringe pumps (two KDS-100 and one KDS-200, Cole-Parmer, Vernon Hills, IL, USA) for precise delivery of the working fluids, a high-voltage power supply (ZGF 60 kV/2 mA, Wuhan Hua-Tian, Wuhan, China) to generate the required electric field, and a grounded collector fabricated by wrapping aluminum foil around rigid cardboard for safety. Four distinct working fluids, whose compositions and operating parameters are listed in Table 1, were selected after preliminary trials. Electrospraying of Fluid 2 yielded microparticles (Figures S1 and S2 in Supplementary Materials), whereas single-fluid electrospinning of Fluid 3 produced nanofibers (Figures S3 and S4 in Supplementary Materials). Process visualization was achieved with a Canon G7X digital camera (Canon, Tokyo, Japan) at various magnifications. Other experimental conditions included: an applied voltage between 13.0 to 15.0 kV to keep a robust and continuous preparation, a fixed fiber collection distance of 20 cm, an ambient temperature of 23 ± 5 °C, and an ambient relative humidity of 49 ± 5%. Both the DEE and the release kinetics strictly depend on the concentration of filler in the matrix [44]; thus, in this study, all three types of nanofibers were prepared from the same drug-loaded working fluids (Fluid 1) under the same fluid flow rate of 3.0 mL/h.

### 2.3. Characterizations of Physical Properties

#### 2.3.1. Morphology

Surface morphology of the three nanofiber types was examined by scanning electron microscopy (SEM; Quanta 450 FEG, FEI, Hillsboro, OR, USA). Prior to imaging, specimens were sputter-coated with platinum for 60 s to ensure adequate conductivity. Fiber diameters and size distributions were subsequently analyzed from the acquired micrographs using ImageJ software V1.8.0 (National Institutes of Health, Bethesda, MD, USA). The applied voltage was 5 keV.

#### 2.3.2. Inner Structure

Transmission electron microscopy (TEM, JEOL, Tokyo, Japan) was employed to probe the internal architecture of the three EC-Cur nanofiber variants. To prepare specimens, a carbon-coated copper grid was positioned directly beneath the spinneret—immediately above the collector—and exposed to the electrospinning jet for approximately 15 s to harvest nanofibers. The settled electron acceleration voltage was 300 keV.

#### 2.3.3. Physical State

A Bruker D8 Advance X-ray diffractometer (Bruker, Ettlingen, Germany) was used to assess the physical form of the raw EC and Cur powders, as well as the physical state of Cur within the three drug-loaded nanofibers (F1, F2, F3). The instrument employed a Cu Kα X-ray source operated at 40 kV and 40 mA. Data were collected from 5° to 40° 2θ with a step size of 0.02°and a scan rate of 10°/min^−1^.

#### 2.3.4. Compatibility

Fourier transform infrared (FTIR) of neat Cur, EC powders, and their electrospun composite nanofibers were collected on a PerkinElmer spectrometer (Billerica, MA, USA). Measurements were performed over 500–4000 cm^−1^ at a spectral resolution of 2 cm^−1^. The KBr tableting method was utilized for preparing all the powder and nanofiber samples.

### 2.4. Characterizations of Functional Performances

#### 2.4.1. Encapsulation Efficiency (DEE %)

A UV–Vis spectrophotometer (model UV-2102C, Unico Instrument Co., Ltd., Shanghai, China) was used for all quantitative analyses of curcumin (Cur). A calibration curve was first constructed with standard solutions to relate absorbance to concentration. To determine the drug encapsulation efficiency (DEE%), Cur was recovered from the nanofibers as follows: (1) An accurately weighed nanofibrous sample was completely dissolved in anhydrous ethanol; (2) 1.0 mL of this ethanolic solution was slowly added to 1000 mL distilled water under vigorous stirring to precipitate the polymer and liberate Cur; (3) After centrifugation (5000 rpm, 10 min), the supernatant was analyzed spectrophotometrically; (4) The Cur concentration in the supernatant was calculated from the established calibration curve, and in turn the mass of Cur measured in the nanofibers (W_m_). DEE % was then computed using Equation (1):

DEE % = (*W*_m_/*W*_p_) × 100 (1)

where *W*_m_ = mass of Cur actually entrapped in the nanofibers (measured experimentally), *W*_p_ = mass of Cur initially added to the working fluid.

#### 2.4.2. In Vitro Release Studies

The release kinetics of Cur from electrospun nanofibers (formulations F1, F2, and F3) were evaluated by the paddle method specified in the Chinese Pharmacopoeia (2020 Edition) as follows: (1) Nanofiber samples equivalent to 50 mg Cur were placed in vessels containing 600 mL phosphate-buffered saline (PBS, pH 7.4), thermostated at 37 °C. The paddles rotated at 50 rpm. (2) At predetermined intervals, 5.0 mL aliquots were withdrawn and immediately replaced with an equal volume of fresh PBS to maintain sink conditions. (3) The absorbance of each sample was measured with the UV–Vis spectrophotometer and converted to concentration (C, μg mL^−1^) using the calibration curve. Cumulative release (%) was calculated with Equation (2):

(2) $$P(\%)=\frac{C_{n}*V_{0}+\sum_{i=1}^{n-1}C_{i}*V}{Q_{0}}*100$$

where *V*_0_ = total dissolution medium volume (600 mL), *V* = sample volume removed (5.0 mL), *Q*_0_ = absolute Cur content in the nanofibers (mg), *C*_n_ = concentration in the nth sample (mg L^−1^), and *Cᵢ* = concentration in the ith sample (mg L^−1^).

### 2.5. Statistical Analysis

All data are reported as mean ± standard deviation (SD). One-way analysis of variance (ANOVA) was employed to detect significant differences among groups. When ANOVA indicated significance (*p* < 0.05), the Dunnett’s post hoc test was applied to identify specific differences.

## 3. Results and Discussion

### 3.1. New EHDA Processes and Their Apparatus

**Figure 1:**
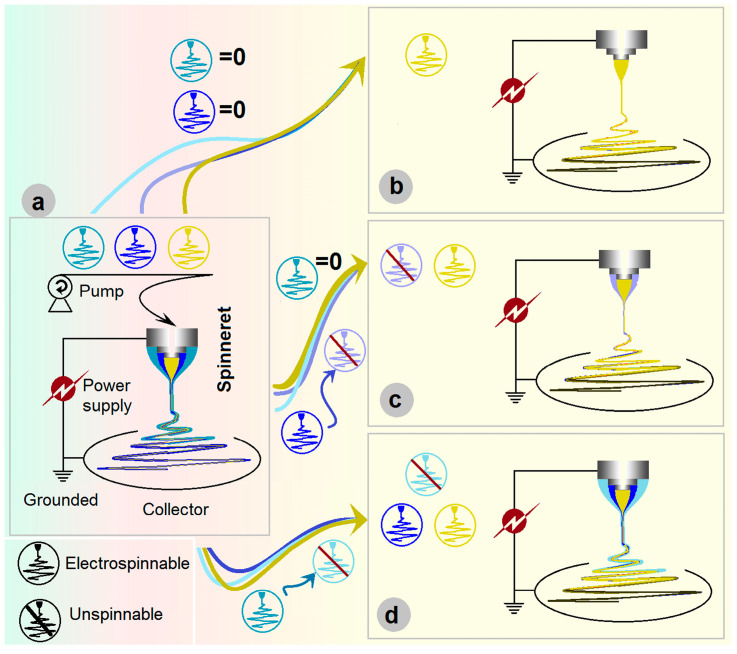
Triaxial electrospinning: (**a**) the parts comprise the triaxial electrospinning apparatus; (**b**) being degraded to a monoaxial single-fluid blending process; (**c**) being degraded to a coaxial process; and (**d**) being changed to a modified triaxial electrospinning with an outer solvent fluid to create double-layer core–sheath nanofibers.

The EHDA apparatus has at least four fundamental parts for initiating an electrospinning process [45,46,47,48,49,50], which include a power supply to provide the high electrostatic voltage, one or more pumps or compressed air for quantitatively driving the working fluids, a collector, and the most important element—spinneret (Figure 1a). For a safe operation, all the elements should be underground during the working processes [51]. An electrospinning process can be termed according to the nozzle’s structure of a spinneret, e.g., a concentric spinneret for a coaxial process [52], an eccentric spinneret for a side-by-side electrospinning process [53], a tri-layer concentric spinneret for a triaxial electrospinning, and a tri-layer eccentric spinneret for a tri-fluid side-by-side electrospinning [54,55].

In the literature, the homogeneous, core–sheath, and tri-layer core–sheath nanofibers are prepared using the monoaxial single-fluid blending electrospinning, coaxial electrospinning, and triaxial electrospinning, respectively. When emulsions were exploited as the treated fluids, the single-fluid blending electrospinning and coaxial electrospinning are able to create core–sheath and tri-layer core–sheath nanofibers [56]. Few reports can be found on the degradation of coaxial electrospinning for creating monolithic nanofibers and the degradation of triaxial electrospinning for producing core–sheath and uniform nanofibers. However, it is obvious that triaxial electrospinning can be facilely degraded into 2-fluid coaxial and 1-fluid blending electrospinning processes simply by switching off one or two of the three working fluids. Shown in Figure 1b–d, the triaxial electrospinning can be degraded to a monoaxial single-fluid blending processes (switching off the middle and outer fluids, Figure 1b), can be degraded to a coaxial process through stopping any one of the three working fluids (Figure 1c), and also can be changed to a modified triaxial electrospinning with an outer solvent fluid to create double-layer core–sheath nanofibers (Figure 1d).

**Figure 2:**
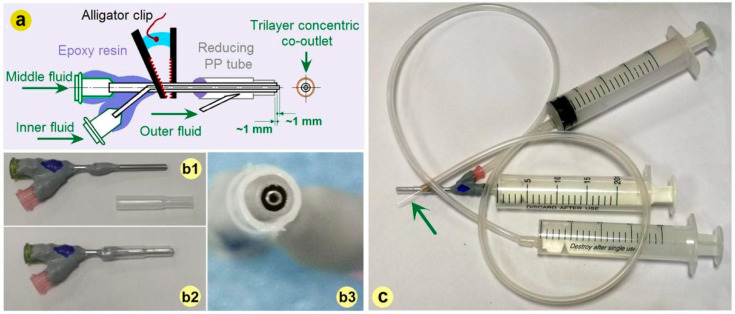
The tri-layer concentric spinneret: (**a**) The inner routes for guiding three fluids from their inlets to the co-outlets; (**b**) The combination of a traditional concentric spinneret prepared from stainless steel capillaries with a PP tubing (from (**b1**) to (**b2**)), a digital image of the tri-layer concentric spinneret’s outlet is shown in (**b3**); (**c**) The connections of spinneret with three syringes for quantitatively transferring the working fluids (the green arrow indicates a very small sharp needle).

The importance of a spinneret not only lies in that the process can be termed according to its nozzle’s structure, but also in that it can be exploited to ensure a continuous and energy-saving process, ensure the production of high-quality nanofibers, and can also be revised conveniently for conducting other electrospinning processes. It is no strange that the spinneret is regarded as the most important element in an electrospinning apparatus and is full of innovations [57,58]. Based on our previous investigations [37], a detachable tri-layer concentric spinneret was developed for conducting the electrospinning processes exhibited in Figure 2.

A diagram about the inner routes for guiding three fluids from their inlets to the co-outlets is given in Figure 2a. Different from our previous spinneret, here the horn tube was replaced by a reducing tube for facile fixing. Its preparation could be facilely realized by covering a traditional concentric spinneret fabricated from stainless steel capillaries with a reducing polypropylene (PP) tube, as indicated by Figure 2(b1,b2). The trilayer concentric co-outlet of the detachable spinneret is exhibited in Figure 2(b3). The connections of the novel spinneret with three syringes for holding and quantitatively transferring the working fluids are shown in Figure 2c. The syringe holding the middle working fluid is directly inserted into the spinneret, and the syringes holding the inner working fluid are connected with the spinneret by a high elastic silicon tube. The syringe holding the outer working fluid is connected with the spinneret by an elastic silicon tube and also a sharp stainless steel needle (as indicated by the green arrow), which can penetrate the PP tube wall easily. For an easy encapsulation and also the prevention of mutual diffusion, the inner, middle, and outer capillaries’ top surfaces are gradually shrunk back about 1.0 mm. The two stainless steel capillaries had outer/inner diameters of 0.6/0.4 mm and 1.5/1.1 mm, respectively. The outlet of the PP tube had an inner and outer diameter of 2.2 mm and 2.8 mm, respectively.

### 3.2. Working Processes

**Figure 3:**
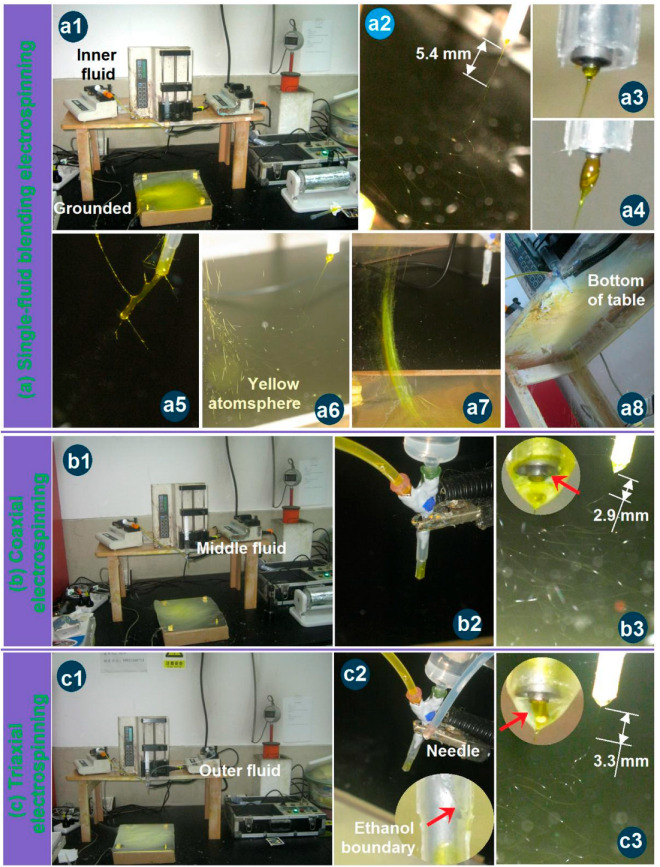
Observations of the different three electrospinning processes: (**a**) The single-fluid blending process from the inner capillary of the tri-layer concentric spinneret for preparing nanofibers F1, the whole apparatus (**a1**), the typical bending and whipping process (**a2**), the typical Taylor cone (**a3**) and its enlargements (**a4**,**a5**), the flying of nanofibers all over the room (**a6**–**a8**); (**b**) The coaxial electrospinning process for producing the nanofibers F2, the apparatus (**b1**), the connections of spinneret with middle and inner fluids and power supply (**b2**); the typical bending and whipping process (**b3**) and the compound Taylor cone (the up-left inset); (**c**) The coaxial electrospinning process for producing the nanofibers F3, the apparatus (**c1**), the connections of spinneret with three fluids and power supply (**c2**); the typical bending and whipping process (**c3**) and the compound Taylor cone (the up-left inset). All the red arrows indicate the fluid-fluid or fluid-air interfaces.

During the three electrospinning processes, a series of interesting phenomena were observed, some of which are captured in Figure 3. As shown in Figure 3a, the single-fluid blending process conducted through the inner capillary of the trilayer concentric spinneret to prepare nanofibers F1 exhibited the most abnormal behavior. The entire apparatus is depicted in Figure 3(a1). Figure 3(a2) presents a typical bending and whipping of the jet that occurred just after the Taylor cone and the straight segment. Based on the spinneret diameter, the length of the straight fluid jet was approximately 5.4 mm. However, Figure 3(a3–a5) reveals that the Taylor cone continuously increased in volume until the process eventually stopped. Consequently, manual removal of the semi-solid substances hanging from the spinneret nozzle was required to allow further conversion of the blended EC-Cur fluid into homogeneous nanofibers. Moreover, the collection of the electrospun nanofibers posed an additional challenge. As illustrated in Figure 3(a6–a8), the solid nanofibers flew throughout the room (Figure 3(a6)), were found suspended between the small support table and the collector (Figure 3(a7)), and even accumulated on the underside of the support table (Figure 3(a8)). Similar manual intervention was necessary during the runs to address these irregularities. These abnormal phenomena associated with the Taylor cone and the collection process exerted pronounced negative effects on the uniformity and quality of the resulting homogeneous EC-Cur nanofibers.

Gupta et al. declared that the sheath working fluid must be electrospinnable for conducting a coaxial electrospinning and a successful preparation of core–sheath nanofibers [59]. Yu et al. broke this traditional concept to carry out a series of modified coaxial electrospinning processes, in which unspinnable fluids, including organic solvents, dilute polymeric solutions, and solutions of small molecules, were exploited as the sheath fluids to create some new kinds of structural nanofibers [60,61]. Based on our previous experiences, when two electrospinnable working fluids are exploited as the sheath and core working fluids, the clogging phenomena are very easy to happen because of the additional interfacial tensions between the sheath and core working fluids. Thus, an unspinnable fluid with an EC concentration of 16% (*w*/*v*) in ethanol was exploited to conduct the coaxial process. The apparatus was the same as the above-mentioned single-fluid blending process, whose whole image is shown in Figure 3b. The only variation was the switching on of the middle working fluid. The connections of the spinneret with the middle and core fluids and the power supply through an alligator clip are shown in Figure 3(b2). Under the optimized electrospinning conditions, a typical coaxial electrospinning process is exhibited in Figure 3(b3). A typical compound inner–outer Taylor cone is given in the upper-left inset, in which the transparent unspinnable EC solution was exploited to encapsulate the core yellow solution (as indicated by the red arrow). However, there were no clear boundaries between them, giving a hint of the diffusion of Cur molecules from the core blended fluid to the middle pure EC solution. By estimation, the straight fluid jet had a length of about 2.9 mm. Compared with the single-fluid electrospinning for preparing nanofibers F1, the length of the straight fluid jet was significantly shortened. The middle unspinnable EC solution was able to end the second step of the straight fluid jet earlier and, in turn, shortened the length of the straight fluid jet. The unspinnable process of EC solution and the optical images of the prepared microparticles are shown in Figure S1 and Figure S2, respectively, in the Supplementary Materials.

For the modified triaxial electrospinning, a spinnable EC solution with a concentration of 25% (*w*/*v*) was exploited as the middle working fluid, but clogging of the spinneret frequently happened (the spinnable and clogging processes of EC solution and the prepared nanofibers are shown in Figure S3 and Figure S4, respectively, in Supplementary Materials). Thus, ethanol was exploited as the outer fluid to prevent clogging for a continuous and robust working process. The whole image is shown in Figure 3c. The connections of the spinneret with three fluids and power supply are given in Figure 3(c2), in which a sharp stainless steel needle penetrated the PP tube’s wall to convert the ethanol to the nozzle of the spinneret through a high elastic silicon tube. The liquid surface of the outer sheath is shown in the bottom-right inset, as indicated by the red arrow. Under the applied electrospinning conditions, a typical triaxial electrospinning process is exhibited in Figure 3(c3). A typical compound trilayer Taylor cone is given in the upper-left inset, in which the transparent ethanol was utilized to encapsulate the middle and core solutions. The middle spinnable EC solution showed a slight yellow color, suggesting some diffusion of Cur molecules from the core fluid to the middle fluid initially at the formation of the Taylor cone. By estimation, the straight fluid jet had a length of about 3.3 mm. Compared with the single-fluid electrospinning for preparing nanofibers F1, the length of the straight fluid jet was significantly shortened in the case of coaxial electrospinning for creating nanofibers F2. The outer ethanol was not only able to prevent the clogging of the spinneret, but also end the second step of the straight fluid jet earlier and, in turn, shorten the length of the straight fluid jet.

### 3.3. Morphologies and Inner Structures

**Figure 4:**
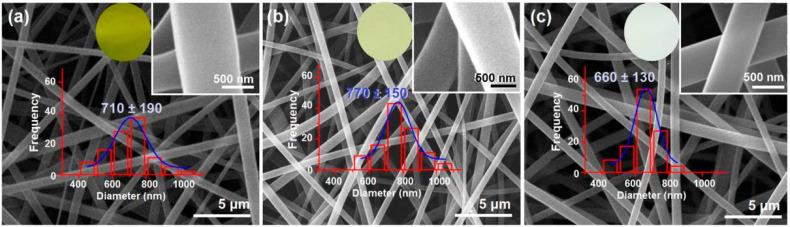
SEM images of EC-based nanofibers: (**a**) blended EC-Cur nanofibers F1 from a single-fluid blending process; (**b**) core–sheath nanofibers F2 from the traditional coaxial electrospinning; (**c**) core–sheath nanofibers F3 from the modified triaxial electrospinning process.

EC has fine electrospinnability [60,62]. As anticipated, all the EC-based nanofibers from the single-fluid blending process, the coaxial process, and the modified triaxial process have the typical linear morphologies, which are exhibited in Figure 4a, 4b, and 4c, respectively. As shown in their upper-right insets, in which a larger magnification was exploited, all these EC nanofibers have a smooth surface. The estimated diameters of the nanofibers F1, F2, and F3 were 710 ± 190 nm, 770 ± 150 nm, and 660 ± 130 nm, respectively. The addition of middle EC fluid should be inclined to increase the nanofibers’ diameters. However, the unspinnable fluid, particularly the outer ethanol in the preparation of nanofibers F3, tended to decrease the nanofibers’ diameters. These two parts co-act to influence the sizes of the resultant nanofibers. Although no trends in the diameter changes, the color changes in these nanofibers are clear, as shown by their optical images in the upper-middle round insets. Nanofibers F1 from the single-fluid blended process had the yellowest color. Due to the blank coating from the unspinnable EC solution, the core–sheath nanofibers F2 had a slightly yellow color, suggesting the encapsulation of Cur molecules in the core sections. As anticipated, the nanofibers F3 had a slightly yellow color, indicating the best sealing effect of the spinnable EC solution on the Cur molecules during the electrospinning process.

**Figure 5:**
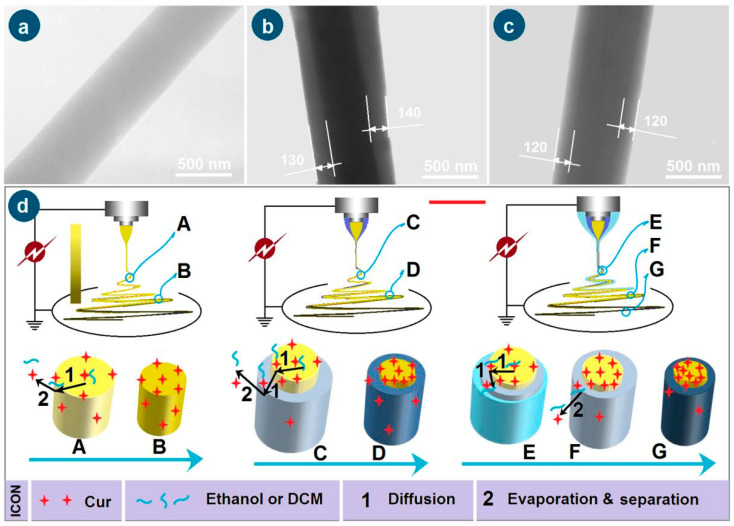
The TEM images of the prepared nanofibers F1 (**a**), F2 (**b**), F3 (**c**), and the mechanisms for elucidating the escapes of Cur molecules from the working fluids to the environments during the various working processes, “1” and “2” show the escape routes of Cur from the working fluids (**d**).

The TEM images of the three types of nanofibers are given in Figure 5a, 5b, and 5c, respectively. The homogeneous nanofibers F1 had the uniform distribution of Cur molecules within the EC matrix, and thus resulted in the uniform gray levels. In contrast, both core–sheath nanofibers F2 and F3 have various regions of their gray levels, as indicated in Figure 5b and 5c, respectively. The sheath sections of F2 and F3 have a thickness of about 130 to 140 nm and 120 nm, respectively. The core sections have higher gray levels due to the following cases: (1) a big thickness of the nanofibers’ central regions; and (2) the loading of Cur molecules, which should fill in the voids of the entanglements of EC molecules, and in turn increase the density of the core sections.

The formation mechanisms of the three working processes, i.e., the single-fluid blending process, the coaxial electrospinning, and the modified triaxial electrospinning, are suggested in Figure 5d. For the preparation of nanofibers F1 through the single-fluid blending electrospinning, the most fundamental mechanism is the continuous drawing of e-jetted fluids to repel out the solvent, leaving the solutes to form the solid nanofibers on the collector during the successive steps, i.e., Taylor cone, straight fluid jet, and the bending and whipping unstable region. The last step contributes mostly to the conversions of working fluids to solid nano products. During these processes, there are two inevitable trends for Cur molecules. One is their escape into the surroundings with the evaporated solvent molecules, which can find the hints in Figure 3(a6), i.e., the slight yellow atmosphere around the heavily yellow fluid jets. This process is diagrammed as “2” in Figure 5d. The other trend is that the Cur molecules are enriched on the surface of fluid jets and solid nanofibers during the continuous drawings and reductions of the size of the jetted fluids/nanofibers, which is shown as “1” in Figure 5d when the fluid jet is changed from “A” to “B”. Although the whole spinning process can be finished on a time scale of several decades of milliseconds, the above-mentioned two trends determine that the drug encapsulation rate within electrospun nanofibers can not be 100% as reported in some literature [37].

When an unspinnable EC solution was exploited as the sheath working fluid to conduct the coaxial electrospinning for creating the core–sheath nanofibers F2, the most fundamental drawing and drying mechanism is modified by the simultaneous traveling of two separate working fluids with an inner-outer relationship. The two trends of direct escape of Cur molecules to the surroundings with the evaporated solvent molecules and the Cur molecules enrichment on the surface of fluid jets and solid nanofibers would be greatly weakened, although there are still very slight yellow atmospheres around the core–sheath compound fluid jets in Figure 3(b3). During the drawing process, the Cur molecules in the inner blended fluid should diffuse partially to the sheath blank EC solution and later go to the surroundings with the repelled out solvent molecules. The key two steps of “1”—diffusion and “2”—evaporation and separation of Cur molecules escape from “C” to “D” are diagrammed in Figure 5d. This should be the reason for observing the slightly yellow at the bottom-left corner of Figure 3(b3).

To further check the feasibility of the hypothesis, a spinnable EC solution but easy to clog spinneret with a concentration of 24% (*w*/*v*) was exploited as a middle working fluid, and pure anhydrous ethanol was exploited as the outer fluid to “lubricate” the working processes for creating the core–sheath nanofibers F3. The most fundamental drawing and drying mechanism is further modified by the simultaneous traveling of three separate working fluids with an inner–middle–outer relationship. On one hand, the outer solvent can act as a “bridge” for the escaping of middle and inner solvent molecules, preventing the possible clogging of semi-substance around the nozzle of the spinneret and retard the formation of semi-solid substance on the surface of fluid jets. On the other hand, the diffusion extents of the Cur molecules in the inner blended fluid to the middle blank EC solution should be weakened. In turn, the above-mentioned two possibilities of direct escape of Cur molecules to the surroundings with the evaporated solvent molecules and the Cur molecules enrichment on the surface of fluid jets and solid nanofibers would be further reduced for achieving a higher DEE value. The two “1”—diffusion steps and the “2”—evaporation and separation step from “E” to “F” and “G” are sketched in Figure 5d.

### 3.4. Physical State and Compatibility

**Figure 6:**
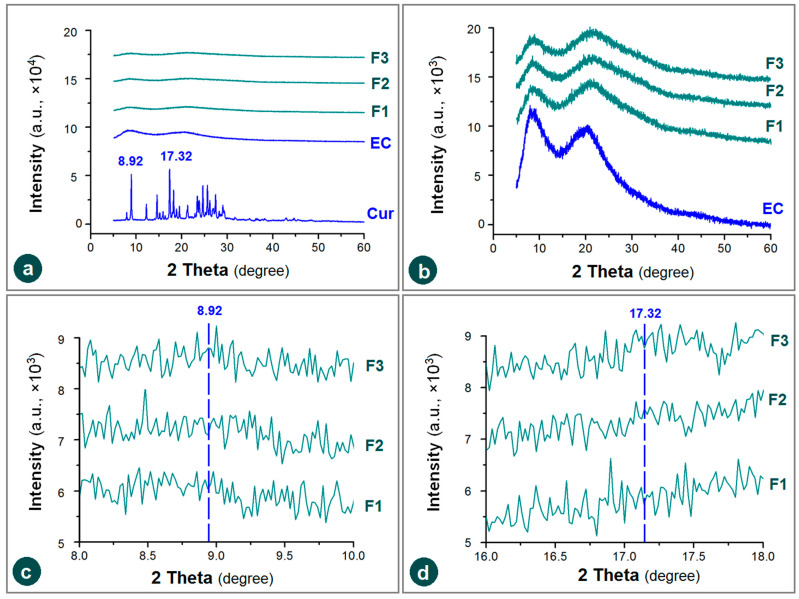
XRD patterns of the raw materials (EC and Cur) and their composite nanofibers F1, F2 and F3 under various *y*-axis scale range: (**a**) a same range of all the materials with the drug; (**b**) a 1/10 smaller y range was utilized to treat the amorphous EC and its composites; (**c**,**d**) the enlargements of *x*-axis to disclose the partial XRD patterns around the sharp peak places of 8.92 degree and 17.32 degree.

XRD patterns are frequently utilized to discern the physical state of drug molecules within their polymeric composites [63]. Many publications have reported that electrospinning can be exploited as a useful method to amorphize the poorly water-soluble drugs, which can be assessed through the absence of sharp Bragg peaks of raw drug crystals [64,65,66]. As anticipated, the raw EC powders, the composite nanofibers F1, F2 and F3 all have a typical pattern without any sharp peaks of the raw Cur powders, e.g., at a place of 8.97 and 17.32 degree, as indicated in Figure 6a. The mission of XRD tests, i.e., to disclose the physical state of drug molecules in the resultant EHDA products (including electrospinning, electrospraying and e-jet printing), is already completed [67,68,69,70,71]. There are no sharp Bragg peaks within the patterns of the several EC-based nanofibers F1, F2 and F3, which appear in the XRD patterns of drug, demonstrated that they were amorphous state with the drug molecules homogeneously distributed among the EC molecules. The electrospinning processes, regardless of the single-fluid blending electrospinning, coaxial electrospinning, or the modified triaxial electrospinning can dry the working fluids within 1 s, propagating the homogeneous distribution state in the working fluids to the solid products. Based on the favorable secondary interactions between the drug and polymer molecules (such as hydrogen bonding and hydrophobic interactions), these electrospun products can present in an amorphous state stably [72,73].

The noises in the XRD patterns of EC and its three medicated fibrous nanocomposites show no significant differences when a similar large *y*-axis scale range was exploited to treat all the involved materials (Figure 6a). When the *y*-axis scale range was reduced 10 times to magnify these XRD patterns, the similarity of patterns and also their noises showed significant differences, as indicated in Figure 6b. The noises on the XRD patterns of amorphous polymers and polymeric composites have no scientific meaning, suggest nothing, form no judgments, and have no influence on the experimental results. They can be even condensed into the smooth lines through setting the scale of *y*-axis (e.g., [74]). Anyway, these noises are unique for each individual XRD pattern, as indicated by the enlargements of *x*-axis scale range to disclose the partial XRD patterns around the sharp peak places of 8.92 degree and 17.32 degree (Figure 6c,d).

**Figure 7:**
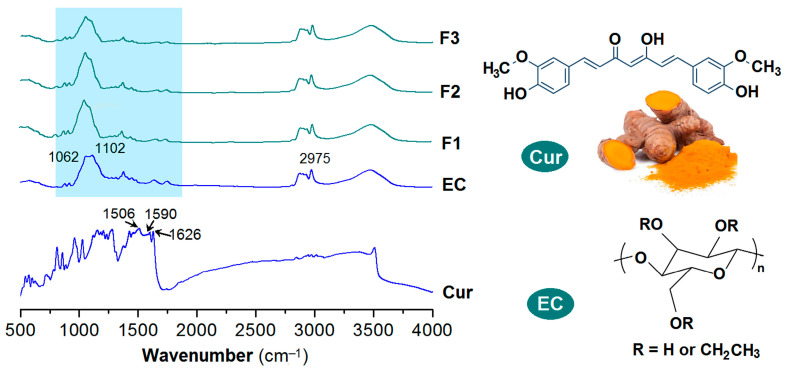
FTIR spectra of the raw materials (EC and Cur) and their composite nanofibers F1, F2, and F3, and the molecular formulas of Cur and EC.

EC is an inert polymeric carrier. It has been exploited to encapsulate many drug molecules and provide them the desired sustained release profiles. The precondition is that these drugs have fine compatibility with EC. Figure 7 (left) displays the FTIR spectra of the individual raw materials—ethyl cellulose (EC) and curcumin (Cur)—along with those of the composite nanofibers F1, F2, and F3. Their corresponding molecular structures are depicted in Figure 7 (right).

EC has the characteristic absorbances at 1062 and 1102 cm^−1^. Cur, extracted from ginger and have a yellow-brown color of its raw powders, contains two benzene rings and other groups including C=O, C=C, and OH groups within one molecule. Its characteristic peaks are at 1506, 1590 and 1626 cm^−1^, which are results from the vibrations of benzene rings and C=C groups. When the Cur molecules were encapsulated into the EC matrices through the single-fluid blending electrospinning, coaxial electrospinning and the modified triaxial electrospinning, the resultant nanofibers F1, F2 and F3 all exhibit an FTIR spectra highly similar with that of EC, regardless of homogeneous nanocomposites or core–sheath heterogeneous nanohybrids. The sharp peaks of raw Cur molecules cannot be clearly discerned out, which should be attributed to the physical secondary interactions between EC and Cur molecules, such as hydrogen bonds between C=O and OH groups, and the hydrophobic interactions between the benzene rings of Cur and the carbon skeleton of EC [37,41] and also the small contents of Cur in the nanofibers. These secondary interactions would make the covalent bonds in the raw crystal powders lose their original vibrations provided the loaded drug content is within a suitable range to avoid phase separation or drug re-crystallization, and thereby the disappearances of the sharp peaks.

### 3.5. Encapsulation Efficiency

**Figure 8:**
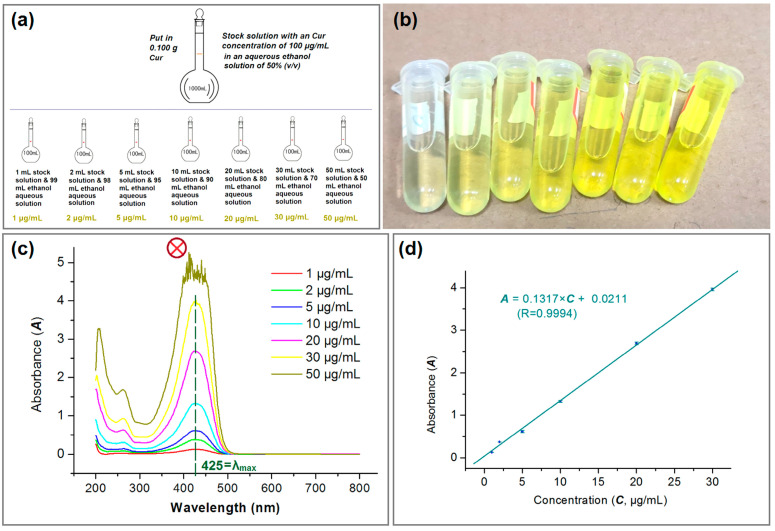
The quantitative UV–vis methods for measuring the concentration of Cur: (**a**) the preparations of a series of solutions with a certain Cur concentration; (**b**) the gradual increase in the yellow color; (**c**) the scanning lines of the various Cur concentration solutions. The symbol of circle with a crossing indicates that this set of data were abandoned for the standard equation; (**d**) the standard equation of Cur (n = 3).

For quantitatively measuring the concentrations of Cur in its solutions, a standard equation reflecting the linear relationship between maximum absorbance at a certain wavelength (*A*) and a certain concentration (*C*) is required. Shown in Figure 8a, a series of standard solutions with a determined concentration of Cur (i.e., 1, 2 5, 10, 20, 30, and 50 μg/mL) were prepared from the Cur stock solution with a concentration of 100 μg/mL in ethanol aqueous solution (with a volume ratio of 50:50). Their yellow colors can be qualitatively discerned in Figure 8b. To scan these standard solutions from 200 to 800 nm, their absorbance curves are collected in Figure 8c. At the concentration of 50 μg/mL, there is no clear single sharp peak at λ_max_ = 425 nm, which should be attributed to its high absorbance and therefore falling outside the linear range of Beer–Lambert’s rule. Thus, the standard equation was built from the six data from 1 to 30 μg/mL, which is expressed as *A* = 0.1317 × *C* + 0.0211 (R = 0.9994). For the measurements of unknown samples, the sample just needed a dilution step and multiplying the reading by the dilution factor provided it was out linear range of 30 μg/mL.

The experimental results indicated that the developed strategy based on the modified triaxial electrospinning process was able to provide a high DEE% of 98.74 ± 6.45%, significantly larger than the values of DEE from the coaxial and blended processes, which were 93.74 ± 5.39% and 88.63 ± 7.36%, respectively. Cur has no sublimation property and has a high boiling point of 593.2 °C. In theory, the DEE% value should be 100% because electrospinning is essentially a rapid physical drying process. However, the measured data are lower than 100%, which aligns with the observations of a slight yellow Cur atmosphere during the preparation of composite nanofibers F1, F2 and F3. During electrospinning, ethanol molecules evaporated into the environment, by which the Cur molecules may follow them to be lost in the environments, thereby, a DEE value smaller than 100% is reasonable. Meanwhile, as diagrammed in Figure 5d, the blank unspinnable EC solutions as the sheath working fluid during the coaxial electrospinning process were useful for preventing the escape of Cur molecules to the environment, and in turn to elevate the values of DEE% from 88.63 ± 7.36% to 93.74 ± 5.39%. To further strengthen the isolation effect between the inner Cur-EC solution with the surroundings, the spinnable EC solutions were exploited as a middle-isolated fluid, and an additional outer ethanol was exploited to lubricate their surfaces for avoiding the clogging of spinneret for creating the core–sheath nanofibers F3, whose DEE% was further elevated to 98.74 ± 6.45%. Thus, the experimental results successfully proved the effectiveness of the developed strategy for ensuring a high DEE% during the electrospinning process.

### 3.6. Sustained Release Profiles

**Figure 9:**
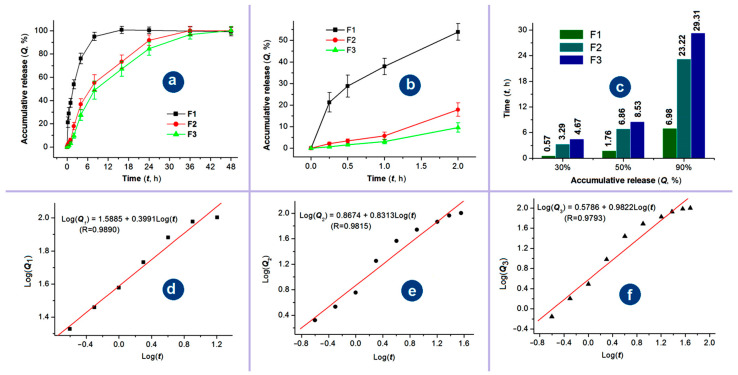
The sustained release profiles of Cur from the three kinds of electrospun medicated nanofibers: (**a**) the whole-range drug in vitro release profiles (mean vlaue ± S.D.); (**b**) the initial release profiles of the nanofibers F1 to F3 at the first 2 h (mean value ± S.D.); (**c**) the time periods for the three types of nanofibers to reach a certain percentage release (30%, 50%, and 90%); (**d**–**f**) the drug release mechanisms of Cur from the three medicated nanofibers, respectively.

Sustained release of the active ingredients can find its applications in a series of scientific and applied fields, such as drug delivery, cosmetics, tissue engineering, active food packaging, agriculture and fertilizer, and general health and care [75,76,77,78,79]. In this study, on the one hand, the sustained release profiles of Cur from the three kinds of electrospun medicated nanofibers over the whole experimental time range are given in Figure 9a. The core–sheath nanofibers F2 and F3 exhibited clearly a better sustained release profile than the homogeneous nanofibers F1 from the blended process with a longer sustained time period. On the other hand, the abnormal initial burst release phenomenon occurred in the curve of nanofibers F1, but not in the curves of nanofibers F2 and F3, as indicated in Figure 9b. Based on the data in Figure 9a,b, the times needed to release a certain percentage of Cur can be calculated through the interpolation method, which are compared in Figure 9c for the percentages of 30%, 50%, and 90%. It is clear that the sustained release time period have an order of triaxial > coaxial > blending, the severity of initial burst release has an order of triaxial ≈ coaxial < blending. Thus, the medicated nanofibers from the three processes have an order of their drug sustained release profiles: triaxial > coaxial > blending in terms of sustained release time periods and also the severe extents of the initial burst release.

EC is an insoluble in water, thus, it can be expected that Cur were released through the molecular penetrations of water molecules from the bulk solutions to the solid nanofibers and later the diffusion of Cur molecules from the nanofibers to the bulk solution. The regressed linear equations between Log(Q) and Log(t) of nanofibers F1, F2 and F3 are shown in Figure 9d, 9e, and 9f, respectively. Based on the achieved n value of 0.3991 (smaller than 0.45), Cur was released from the blended nanofibers F1 through the typical Fickian diffusion mechanism as expected. However, the regressed n values of the core–sheath nanofibers F2 and F3 were 0.8313 and 0.9822, respectively. These unexpected results should be attributed to the precondition of the Peppas equation, which assumes that drug molecules are homogeneously distributed within the polymeric matrices [80,81]. In the cases of nanofibers F2 and F3, the Cur molecules were only distributed within the core sections and were completely covered by a blank EC sheath layer. It is just the blank layers; the exponent of the regression equation was unreliable for determining the Cur release mechanism in the current investigations. Cellulose and its derivatives are highly popular in drug delivery applications because they can provide a wide range of controlled-release profiles—including rapid, sustained, and multi-phase release. This versatility stems from their excellent processability, biocompatibility, stability, tunable swelling behavior, and favorable wet deformability [82,83].

### 3.7. Mechanisms of Sustained Release and the Related Process-Structure-Performance Relationship

**Figure 10:**
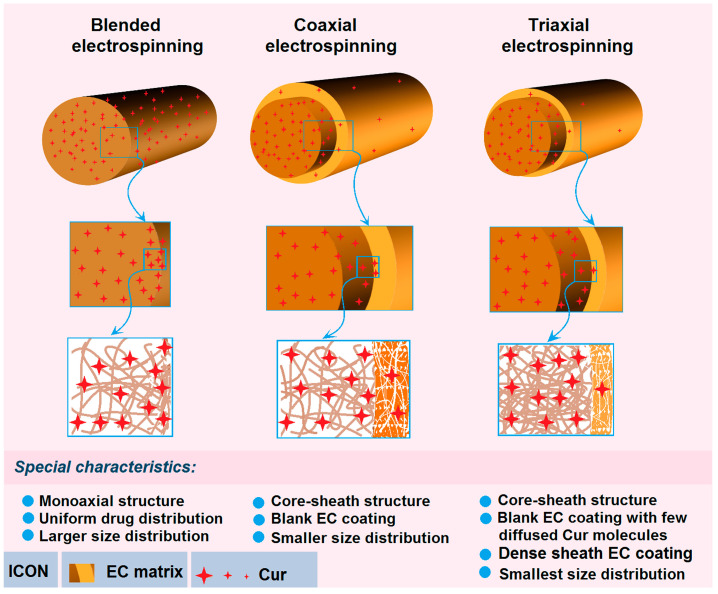
The drug sustained release mechanisms from the three kinds of medicated nanofibers and the related process–structure–performance relationship.

Shown in Figure 10 are the diagrams about the sustained release mechanisms of Cur molecules from their three kinds of medicated nanofibers fabricated using the single-fluid blending process, coaxial process and the modified triaxial process. For nanofibers F1, they have a typical monoaxial structure with all the Cur molecules uniformly distributed all over the EC matrices. These structural characteristics determine that there are abundant Cur molecules on the large surface of the nanofibers. These Cur molecules can be directly dissolved into the dissolution media when the nanofibers are placed into the dissolution vessels. Thus, the initial burst release is inevitable for the blended medicated nanofibers. Later, the Cur molecules are released in a more and more “difficult” manner. One reason is the diffusion routes for both the penetration of water molecules and also the Cur molecules from the nanofibers to the dissolution media. The other is that the receded surfaces contain fewer and fewer Cur molecules. What is more, these nanofibers have a poor size distribution, which would further spoil the stable and sustained release properties.

When the medicated nanofibers F2 are coated by a blank sheath layer of EC through the coaxial electrospinning, the structural nanofibers have almost no Cur molecules on their surfaces except a few Cur molecules diffused into the sheath sections during the bending and whipping processes. Meanwhile, the unspinnable EC solution can ensure a more robust and continuous working process, and in turn result in nanofibers with a more uniform size distribution. These factors are able to co-act to eliminate completely the initial burst release phenomena, to prolong the sustained release time period due to the intentional increase in diffusion routes of Cur molecules when they are dissolved by the penetrated water molecules, diffused from the core sections of nanofibers to the outer dissolution media through the sheath blank EC barrier layer.

However, when a pure ethanol is exploited as an outer fluid and a spinnable EC solution is exploited as the middle fluid to increase the encapsulation effects, the resultant core–sheath nanofibers F3 have some advantages over their peers from the traditional coaxial process in the following aspects: (1) the sheath blank EC coating has fewer Cur molecules diffused from the inner blended solution due to the higher EC concentration and the larger viscosity; (2) the denser sheath section due to the outer ethanol can prevent the previous formations of semi-solid substance to let the exhaustion of ethanol molecules in the middle and also inner working fluids; and (3) the outer ethanol can ensure a longer time period of drawing and the stability of electrospinning processes, and in turn their smallest size distribution. These favorable factors can make the core–sheath nanofibers F3 further improve the sustained release profiles with an even larger DEE% value. Thereby, the modified triaxial electrospinning can be exploited to provide the best process–structure–performance relationship for the sustained release of Cur molecules from the EC matrices.

New techniques for efficient materials conversion are in high demand across many scientific disciplines. Reported modified tri-axial electrospinning possesses several unique advantages over conventional electrospinning: (1) it enables continuous, robust operation free of clogging or other anomalies; (2) it can process otherwise unspinnable fluids, thereby expanding the range of polymers that can be converted into nanofibers; (3) it allows facile manipulation of core–shell nanostructures; and (4) it reliably maintains the desired process–structure–performance relationship. Building on the broad applicability of blending electrospinning [84,85], and through judicious selection of polymeric matrices and compatible drugs [82,83,86], modified tri-axial electrospinning is expected to open new avenues in pharmaceutics. Moreover, when combined with advanced tools such as molecular simulation, AI, and electrospraying [87,88], this technique can yield even more sophisticated nanomaterials and novel insights.

## 4. Conclusions

A modified triaxial electrospinning protocol was devised in which a viscous EC solution served as the intermediate layer to isolate an inner Cur-loaded fluid. This high-EC middle layer effectively blocks outward diffusion of Cur during jet formation. A third, outermost shell of pure ethanol was introduced to lubricate the spinneret, eliminating clogging and allowing uninterrupted fabrication of core–sheath nanofibers F3. Performance comparisons illustrate the benefits of this architecture: (1) Versus homogeneous (F1) and traditional coaxial (F2) fibers, F3 achieved a Cur DEE%; (2) The core–sheath geometry suppressed the initial burst release common to F1 and F2, extending the sustained-release time periods. The formation mechanism of the tri-layer jet is discussed in detail, together with how the core–sheath geometry modulates drug diffusion. The work thereby establishes a clear process–structure–performance linkage and offers a general blueprint: an auxiliary solvent stream can be added to existing electrospinning setups to create more sophisticated nanostructures with improved or entirely new functionalities.

## Figures and Tables

**Table 1:** The parameters for creating nanofibers using three electrospinning processes.

No.	Working Process	Working Fluid/Flow Rate (mL/h)			Cur Content ^a^ (%)
		Outer	Middle	Inner	
F1	Single-fluid blending	----	----	Fluid 1/3.0	11.1%
F2	Coaxial	----	Fluid 2/1.2	Fluid 1/3.0	9.0%
F3	Modified triaxial	Ethanol/0.5	Fluid 3/0.8	Fluid 1/3.0	9.0%

^a^ Theoretic value calculated according to the experimental conditions. Fluid 1: EC solution comprised 24.0 g EC and 3.0 g Cur in 100 mL ethanol/DCM mixture (50:50 in volume). Fluid 2: EC solution consisted of 16.0 g EC in 100 mL ethanol/DCM mixture (50:50 in volume). Fluid 3: EC solution consisted of 24.0 g EC in 100 mL ethanol/DCM mixture (50:50 in volume).

## Data Availability

The data supporting the findings of this manuscript are available from the corresponding authors upon reasonable request.

## Supplementary Materials

The following supporting information can be downloaded at: https://www.mdpi.com/article/10.3390/pharmaceutics17091152/s1, Figure S1: The electrospraying process of the unspinnable EC fluid, the time cost from (a) to (d) is about 5 minutes. Figure S2: Optical images of the collected EC particles on the glass slide prepared from the unspinnable fluid 2 (under a magnification of 20 × 40). Figure S3: The electrospinning process of the spinnable EC fluid 3, the time cost from (a) to (d) is about 3 min. Figure S4: Optical images of the collected EC nanofibers on the glass slide prepared from the electrospinnable fluid 3 (under a magnification of 20 × 40) [22,88,89,90,91,92].
