# Supplementary material for: A Modified Triaxial Electrospinning for a High Drug Encapsulation Efficiency of Curcumin in Ethylcellulose
Source: Pharmaceutics. 2025 Sep 2;17(9):1152. doi: 10.3390/pharmaceutics17091152 (PMC12473905; doi:10.3390/pharmaceutics17091152)
Supplement: Supplementary file 1 [file pharmaceutics-17-01152-s001.zip › pharmaceutics-3786226-supplementary.pdf]

Supplementary Materials

A Modified Triaxial Electrospinning for A High Drug Encapsulation Efficiency of Curcumin in Ethylcellulose

Xingjian Yang,[1,†] Qiling Wang,[1,†] Zhirun Zhu,[2] Yi Lu,[1] Hui Liu,[1] Deng-Guang Yu,[1,*] Sim-Wan Annie Bligh[3,*]

[1] School of Materials and Chemistry, University of Shanghai for Science and Technology, 516 Jungong Road, Shanghai 200093, PR China
[2] Shanghai Experimental School, 300 Dongming Road, Shanghai, 200125, China
[3] School of Health Sciences, Saint Francis University, Hong Kong 999077, China
Correspondence: ydg017@usst.edu.cn (D.-G. Yu); abligh@sfu.edu.hk (S.W.A. Bligh)
[†] These authors contributed equally to this work.

S1. Experiments

A homemade electrospinning apparatus was set up for testing the electrospinnability of various working fluids, which comprised a spinneret (a stainless steel capillary with an inner diameter of 0.51 mm), a pump (KDS 100, Cole-Parmer, USA), a high power supply (ZGF60kV/2mA, Wuhan Hua-Tian High Power Co., Ltd., Wuhan, China) utilized to provide the high electrostatic voltages, and a simple collector made by wrapping aluminum foil around a hard cardboard. Both the power supply and fiber collector were grounded for safety. A Canon G7X digital camera from Canon Corporation (Tokyo, Japan) was used to observe the electrospinning processes with various magnifications.

The details about the two fluids for testing electrospinnability are listed as follows:

Unspinnable EC fluid 2: EC solution consisted of 16.0 g EC in 100 mL ethanol /DCM mixture (50:50 in volume).

Electrospinnable EC fluid 3: Fluid 3: EC solution consisted of 24.0 g EC in 100 mL ethanol /DCM mixture (50:50 in volume)..

S2. Characterizations

An optical microscopy (XP100, Shanghai Changfang Optical Instrument Co., Ltd., Shanghai, China) was utilized to observe the morphologies of the resultant EHDA products. The sampling was conducted by placing a glass slide just under the spinneret's nozzle but above the collector for about 10 minutes. Then, the glass slide was placed under the optical microscope to observe at a suitable magnification.

S3. Results and discussion

S3.1. Electrospraying of the unspinnable EC Fluid 2

As indicated in Figure S1, the unspinnable EC fluid F2 had no electrospinnability due to no enough physical entanglements to resist the electrical forces [S1-S3] within the EC solution when its concentration was only 16% (w/v). The typical three steps, i.e. the Taylor cone, the convergent point, and the followed atomization are obvious [S4, S5]. However, the Taylor cone continuously increased its volume. The time cost from (a) to (d) is about 5 minutes. The reasons should be attributed to the easy evaporation of DCM and ethanol, the easy formation of semi-solid substance on the surface of fluid jets, and the adhesive property of EC solutions on the surface of the stainless steel capillaries. The optical images of the resultant ultra-thin particles are shown in Figure S2.

The electrospinning processes of the spinnable EC fluid 3 are shown in Figure S3, the time cost from (a) to (d) is about 3 minutes. The optical images of the resultant ultra-thin nanofibers are shown in Figure S4. EC and some other cellulose-based derivatives have fine electrospinnability [S6]. However, high volatile organic solvents are frequently exploited to dissolve them, which also comprises an important reason to the frequent clogging phenomena.

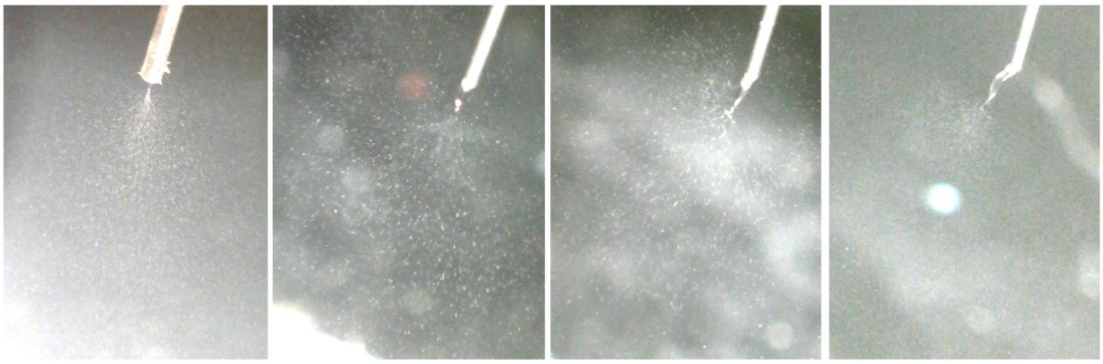

(a) (b) (c) (d)

Figure S1: The electrospraying process of the unspinnable EC fluid, the time cost from (a) to (d) is about 5 minutes.

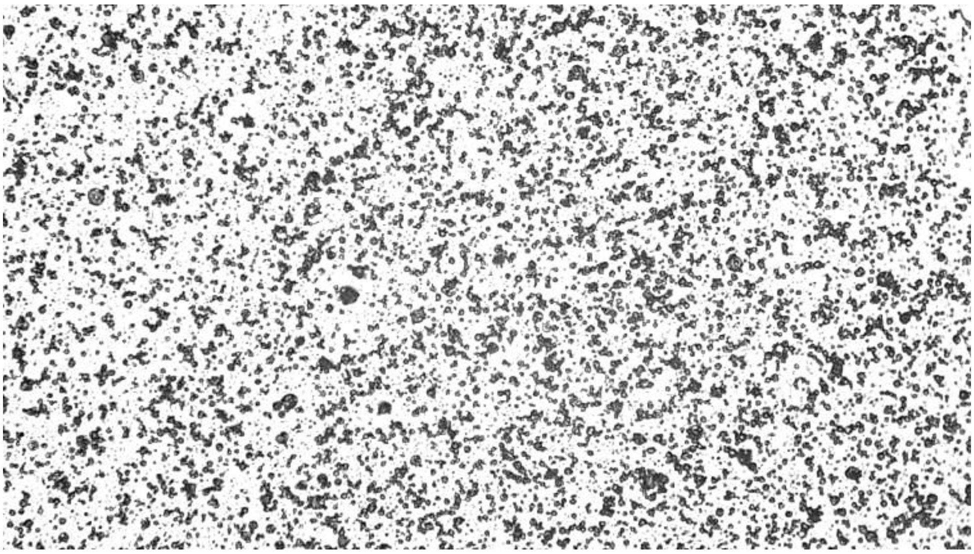

Figure S2: Optical images of the collected EC particles on the glass slide prepared from the unspinnable fluid 2 (under a magnification of 20×40).

S3.2. Electrospinning of the spinnable EC Fluid 3

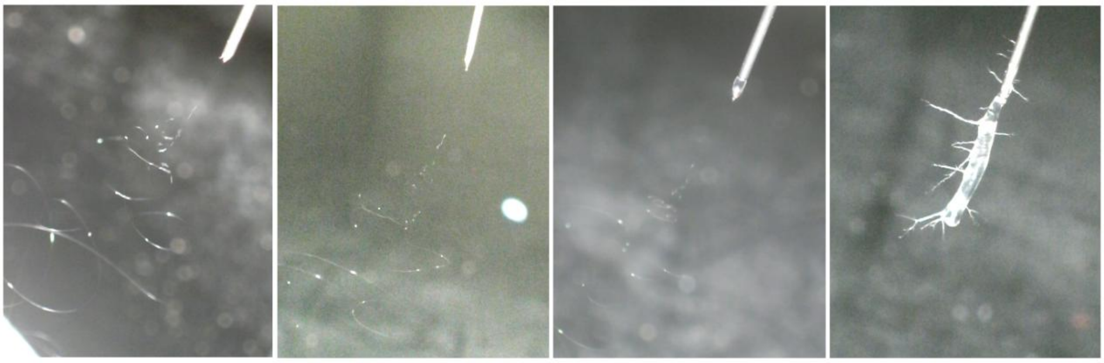

(b) (b) (c) (d)

Figure S3: The electrospinning process of the spinnable EC fluid 3, the time cost from (a) to (d) is about 3 minutes.

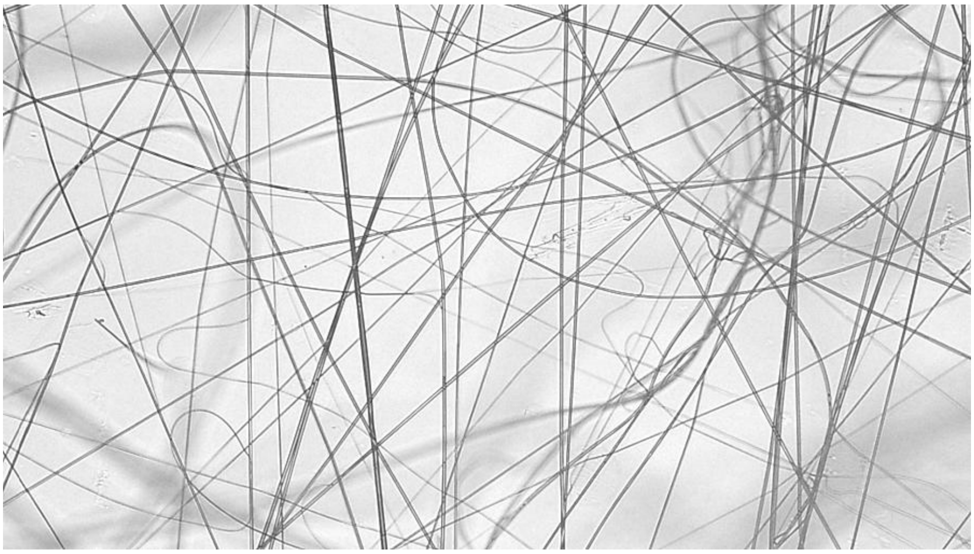

Figure S4: Optical images of the collected EC nanofibers on the glass slide prepared from the electrospinnable fluid 3 (under a magnification of 20×40).

References

S1. Li, D.; Yue, G.; Li, S.; Liu, J.; Li, H.; Gao, Y.; Liu, J.; Hou, L.; Liu, X.; Cui, Z.; et al. Fabrication and Applications of Multi-Fluidic Electrospinning Multi-Structure Hollow and Core–Shell Nanofibers. *Engineering* **2022**, *13*, 116–127. doi:10.1016/j.eng.2021.02.025.

S2. Brimo, N.; Serdaroğlu, D.Ç.; Uyar, T.; Uysal, B.; Çakıcı, E.B.; Dikmen, M.; Canturk, Z. Novel Electrospun Polymeric Nanofibers Loaded Different Medicaments as Drug Delivery Systems for Regenerative Endodontics. *Curr. Drug Deliv.* **2023**, *20*, 992–1014. doi:10.2174/1567201819666220418102732.

S3. Bayer, I.S. Controlled Drug Release from Nanoengineered Polysaccharides. *Pharmaceutics* **2023**, *15*, 1364. doi:10.3390/pharmaceutics15051364.

S4. Chen, S.; Wu, X.; Ding, X. One-Step Side-by-Side Electrospraying of Janus Particles for Durable Multifunctional Coatings on Cotton Textiles. *Colloid. Surf. A* **2025**, *710*, 136227. https://doi.org/10.1016/j.colsurfa.2025.136227.

S5. Chen, S.; Zhou, J.; Fang, B.; Ying, Y.; Yu, D.G.; He, H. Three EHDA Processes from a Detachable Spinneret for Fabricating Drug Fast Dissolution Composites. *Macromol. Mater. Eng.* **2024**, *309*, 2300361. doi:10.1002/mame.202300361.

S6. Deng, Y.; Zhu, T.; Cheng, Y.; Zhao, K.; Meng, Z.; Huang, J.; Cai, W.; Lai, Y. Recent Advances in Functional Cellulose-Based Materials: Classification, Properties, and Applications. *Adv. Fiber Mater.* **2024**, *6*, 1343-1368. doi:10.1007/s42765-024-00454-0.
